# **γ**-Secretase Pharmacology: What Pharmacology Will Work for Alzheimer's Disease?

**DOI:** 10.1155/2013/849128

**Published:** 2013-04-18

**Authors:** Jeremy H. Toyn, Adele Rowley, Yasuji Matsuoka, Taisuke Tomita, Bruno P. Imbimbo

**Affiliations:** ^1^Neuroscience Biology, Bristol-Myers Squibb Research and Development, 5 Research Parkway, Wallingford, CT 06492, USA; ^2^TopiVert Pharma Ltd., Imperial College Incubator, Bessemer Building Level 1, Imperial College, London SW7 2AZ, UK; ^3^Neural Pathways Discovery, GlaxoSmithKline, Singapore Research Center, Singapore 138667; ^4^Department of Neuropathology and Neuroscience, Graduate School of Pharmaceutical Sciences, The University of Tokyo, Tokyo 113-0033, Japan; ^5^Research & Development, Chiesi Farmaceutici, Largo Francesco Belloli, 11/a, 43122 Parma, Italy

This special issue focuses on *γ*-secretase modulators (GSMs) and inhibitors (GSIs), two classes of small molecules with the potential to test the amyloid hypothesis of Alzheimer's disease. Recent clinical trials of GSI and GSM, including semagacestat, avagacestat, and R-flurbiprofen, have been discontinued for lack of efficacy and/or side effects, the mechanisms of which have not been elucidated. Detrimental effects of GSIs on cognition observed in AD patients may be linked to the accumulation of C-terminal fragment of APP (C99 or CTF*β*). The stimulating effects of GSIs on skin cancer in AD patients have been linked to their inhibition of Notch processing. The lack of efficacy of the GSM R-flurbiprofen in AD patients has been explained with its low potency and poor ability to cross the blood-brain barrier. The two review articles and three research articles address key issues for GSI and GSM, namely, Notch-related side effects and drug-like properties, respectively. Although other amyloid-related approaches are continuing in clinical trials, including anti-A*β* antibodies and *β*-site amyloid precursor protein cleaving enzyme (BACE) inhibitors, it still remains to be seen whether or not they can decrease amyloid or A*β* for a sufficient period of time at tolerable doses in patients. Therefore, renewed efforts toward GSIs and GSMs appear justified.

The review by M. Takami and S. Funamoto provides a succinct but comprehensive introduction to the stepwise proteolytic mechanism of *γ*-secretase. They describe the experimental evidence for the multiple cleavages of the amyloid precursor protein (APP) and provide some particularly useful illustrations showing how the enzyme processes its substrates to make a series of A*β* peptides and tripeptides. They further propose that *γ*-secretase cleavage of Notch may be mechanistically different from the cleavage of APP, implying an untapped potential to discover APP-selective or Notch-selective inhibitors.

H. J. M. Gijsen and M. Mercken review the current status of GSM drug discovery, identify the limitations of current molecules, and present an approach to the future optimization of GSMs. The benchmark GSMs are succinctly reviewed and common trends are evaluated. They give a thoughtful and accessible explanation of the “conflict between the physicochemical properties required for highly efficacious GSM and those (properties) required for drug-likeness.” This contrasts with GSI, where high potency and optimal physical properties have been achieved for some molecules, but the biological mechanism imposes inbuilt Notch-related side effects. Thus, benchmark GSMs suffer from low quality physical properties, whereas benchmark GSIs are of high drug-like quality but suffer from a limitation of the biological mechanism. The quantitative use of biological and physical properties in drug optimization is reviewed in a straightforward way, and illustrations are used to show where the current GSM and GSI molecules stand in this quantitative analysis. Also included is new data relating to a novel GSM illustrating recent progress.

B. Tate et al. review the benchmark GSIs and GSMs while providing data describing the novel natural product-derived GSMs discovered by Satori. GSMs typically decrease A*β*42 and increase A*β*38 production; however, the Satori compounds decrease both A*β*42 and A*β*38, while increasing A*β*37 and A*β*39. These different effects on A*β* peptides are well illustrated by straightforward MALDI-TOF scans. An expanded definition of GSM is therefore given as compounds that cause “a shift of the A*β* pool to shorter, but variable length, A*β* peptides.” In contrast to GSI, for which potency is sensitive to APP expression level, GSMs exhibit a relatively constant level of potency. Nevertheless, GSMs require high plasma concentrations for associated brain A*β* lowering, an observation that cannot be fully explained by properties such as nonspecific protein binding and brain penetrance.

The original research article by L. A. Hyde et al. describes approaches to mitigate the risk of Notch-related toxicity in rodents given the GSI SCH697466. Notch-related side effects were evaluated in the intestine and thymus, and Notch-related biomarkers were monitored in white blood cells. Either decreased frequency of dosing, or lower doses which caused a partial lowering of A*β*, were shown to decrease Notch inhibition. The results show that appropriate choices of the extent and duration of dosing can facilitate significant A*β* lowering without evidence of Notch-related side effects.

In the original research article by M. C. de Vera Mudry et al., the GSI RO4929097 was given to rats and mice to explore the effect on immune responses. One of the most characteristic Notch-related side effects of GSI is spleen marginal zone atrophy, which raises the possibility of a consequence for immune responses. Remarkably, despite the atrophy and decreased B cell numbers caused by chronic dosing of the GSI, the effect on immune responses was mild and reversible.

In summary, the main theme of this special issue is about recent approaches to address the limitations of the two main classes of *γ*-secretase-targeted small molecules. For GSIs, the focus is on the mitigation of side effects related to Notch and other non-APP substrates. Extent and duration of GSI dosing could be varied to decrease effects on Notch, and the consequences of decreased B cell numbers on immune responses were shown to be mild, thereby diminishing a perceived risk. Furthermore, the mechanisms of the *γ*-secretase cleavages of APP and Notch, while not well understood, may be sufficiently different to facilitate improved APP- or Notch-selectivity of GSIs in the future. For GSMs, the focus is on improving the drug-like physicochemical properties while maintaining high potency. The future of GSMs therefore appears to be a continuation of a challenging multiparameter drug optimization. We hope readers will derive intellectual benefit from these papers and enjoy them as much as we did.

*Jeremy H. Toyn* *Jeremy H. Toyn* *Adele Rowley* *Adele Rowley* *Yasuji Matsuoka* *Yasuji Matsuoka* *Taisuke Tomita* *Taisuke Tomita* *Bruno P. Imbimbo* *Bruno P. Imbimbo*

